# Supplementary material for: GDAP1 Involvement in Mitochondrial Function and Oxidative Stress, Investigated in a Charcot-Marie-Tooth Model of hiPSCs-Derived Motor Neurons
Source: Biomedicines. 2021 Aug 2;9(8):945. doi: 10.3390/biomedicines9080945 (PMC8393985; doi:10.3390/biomedicines9080945)
Supplement: Supplementary file 1 [file biomedicines-09-00945-s001.zip › Supplementary Materials - F Miressi.pdf]

Supplementary Materials

Supplementary Tables

Table S1. Primary antibodies used for characterization of hiPSCs, fibroblasts, and neural cells.

Antibody	Company	Cat. Number	Species	Dilution
Pluripotency markers				
**Sox2**	Biolegend	630802	Rabbit	1:200
**Oct3/4**	Santa Cruz Biotech	sc-5779	Mouse	1:50
**Nanog**	DSHB	PCRP-NANOGP1-2D8-s	Mouse	1:5
Germinal Layers markers				
**α-SMA**	DAKO	M0851	Mouse	1:500
**Sox17**	R&D	AF1924	Goat	1:100
**MAP2**	Merck	M-4403	Mouse	1:500
Neural markers				
**PGP9.5**	Abcam	ab108986	Rabbit	1:100
**Tuj1**	R&D	MAB1195	Mouse	1:1000
**Chat**	Chemicon	AB144P	Goat	1:50
Other				
**GDAP1**	Proteintech	13152-1-AP	Rabbit	1:100
**P4HB**	OriGene	AF0910-1	Mouse	1:100
**Ki-67**	Leica	NCL-L-Ki67-MM1	Mouse	1:100

Sanger sequencing

Genomic DNA was extracted from fibroblasts of Ctrl-1, Ctrl-2, and patient, using the Puregene Tissu kit (©QIAGEN), and following manufacturer's instructions. Sanger sequencing was performed for all *GDAP1* exons. It confirmed, in patient's DNA, the presence of the homozygous c.581C>G mutation in exon 5 of *GDAP1*, responsible for the amino acidic substitution of the Serine 194 (TCA) with a stop codon (TGA), in GDAP1 protein. No *GDAP1* mutation was detected in Ctrl-1 and Ctrl-2 (Figure S1).

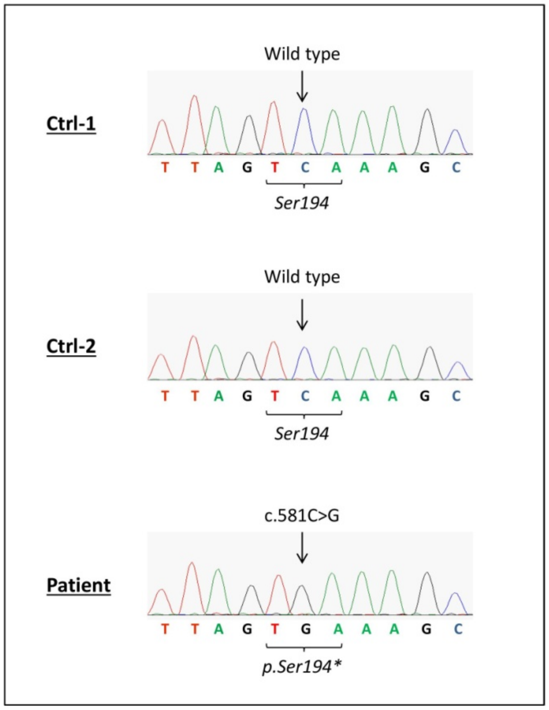

Figure S1. Sanger Sequencing confirmed the *GDAP1* homozygous c.581C>G mutation in patient's fibroblasts, not observed in controls.

hiPSCs generation, maintaining, and validation

Following the iStem (INSERM/UEVE UMR861, AFM, Genopole, Evry, France) procedure, hiPS cells were generated from fibroblasts of Ctrl-1 (Figure S2A), Ctrl-2 (Figure S2B), and the CMT-patient (Figure S2C). Two weeks after fibroblasts' nucleofection, hiPSCs colonies were selected for each subject. One clone for each subject was amplified and, at passage 15, validated with all quality controls. Colonies' morphology was checked. Pluripotency was established by the alkaline phosphatase test, the EB formation, the spontaneous differentiation in the three germ layers, and immunocytochemistry analysis for pluripotency markers (Figure S2). We verified also, by array Comparative Genomic Hybridization (aCGH), the lack of large genomic copy number variations (CNVs) in hiPSCs genome. Thus, for Ctrl-1, Ctr-2, and the patient, aCGH allowed comparing the genome of hiPSCs with the genome of own fibroblasts. It demonstrated the absence of novel CNVs, as large deletions or duplications, supporting that hiPSCs and fibroblasts have the same genetic background (data not shown).

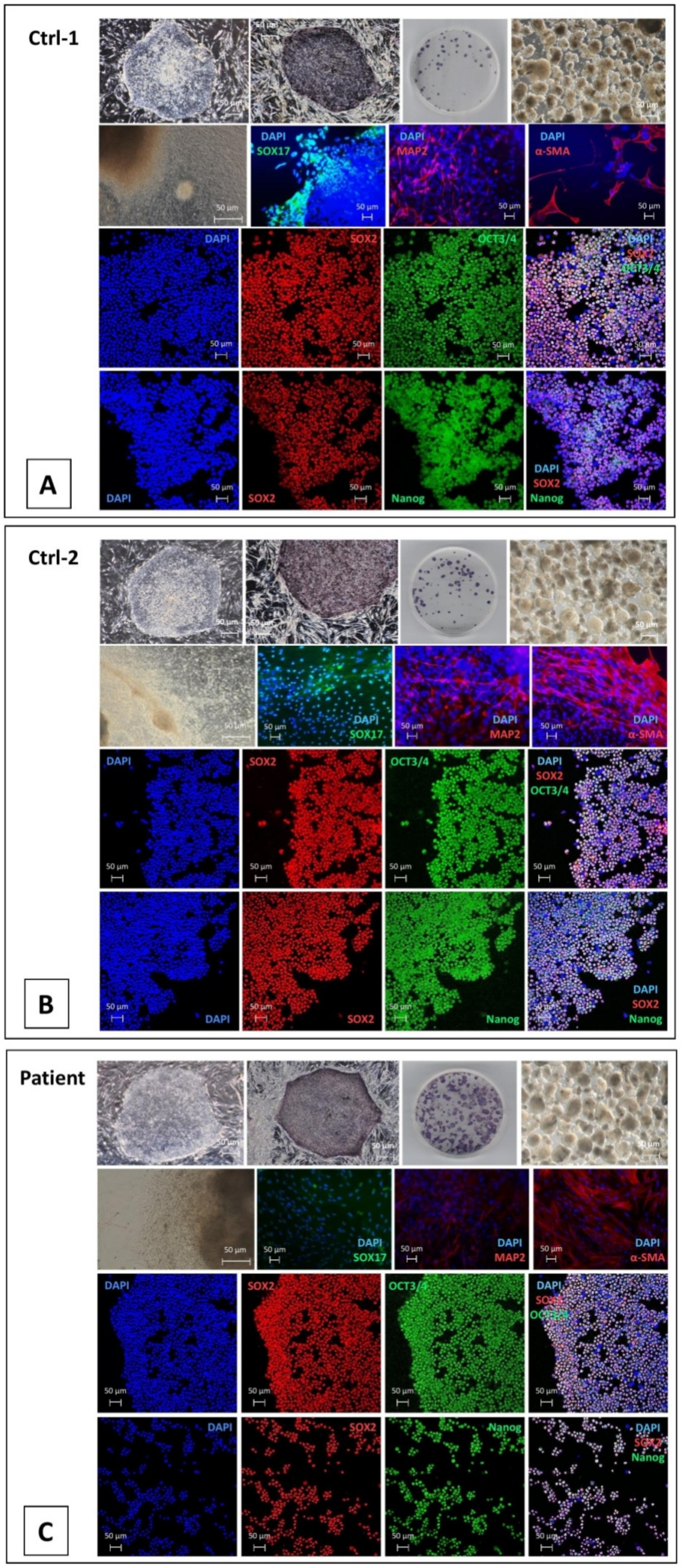

Figure S2. HiPSCs quality controls for Ctrl-1 (**A**), Ctrl-2 (**B**), and patient (**C**). hiPSCs colonies had a typical morphology, expressed alkaline phosphatase, could originate EB, and spontaneous differentiate in the three embryonic germ layers [Sox17 (green): endoderm, α-SMA(red): mesoderm, MAP2 (red): ectoderm]. hiPSCs expressed also pluripotency markers: Oct3/4 (green), Sox2 (red), Nanog (green). Scale bar = 50 µm.

Motor neurons generation and validation

For each subject, hiPSCs were led to neural differentiation, following the protocol described by Faye *et al* [28]. Briefly, hiPSCs colonies were cut to obtain EB, which were first cultured in suspension, and secondly seeded to generate rosettes. The selection of rosettes allowed isolating neural progenitors (NPs), which were amplified and, after 5-6 passages, seeded at low density, to generate motor neurons (MNs). The same protocol was successfully performed for Ctrl-1, Ctrl-2, and the CMT-patient (Figure S3).

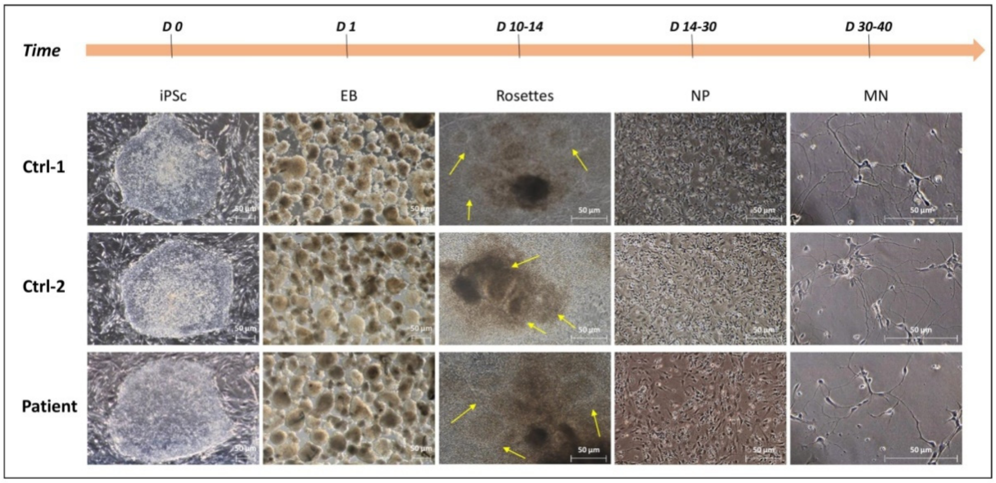

Figure S3. hiPSCs differentiation into motor neurons, for Ctrl-1, Ctrl-2 and patient from D0 to D30-40. Scale bar = 50 μm.
