# Supplementary figures and images for: GDAP1 Involvement in Mitochondrial Function and Oxidative Stress, Investigated in a Charcot-Marie-Tooth Model of hiPSCs-Derived Motor Neurons
Source: Biomedicines. 2021 Aug 2;9(8):945. doi: 10.3390/biomedicines9080945 (PMC8393985; doi:10.3390/biomedicines9080945)

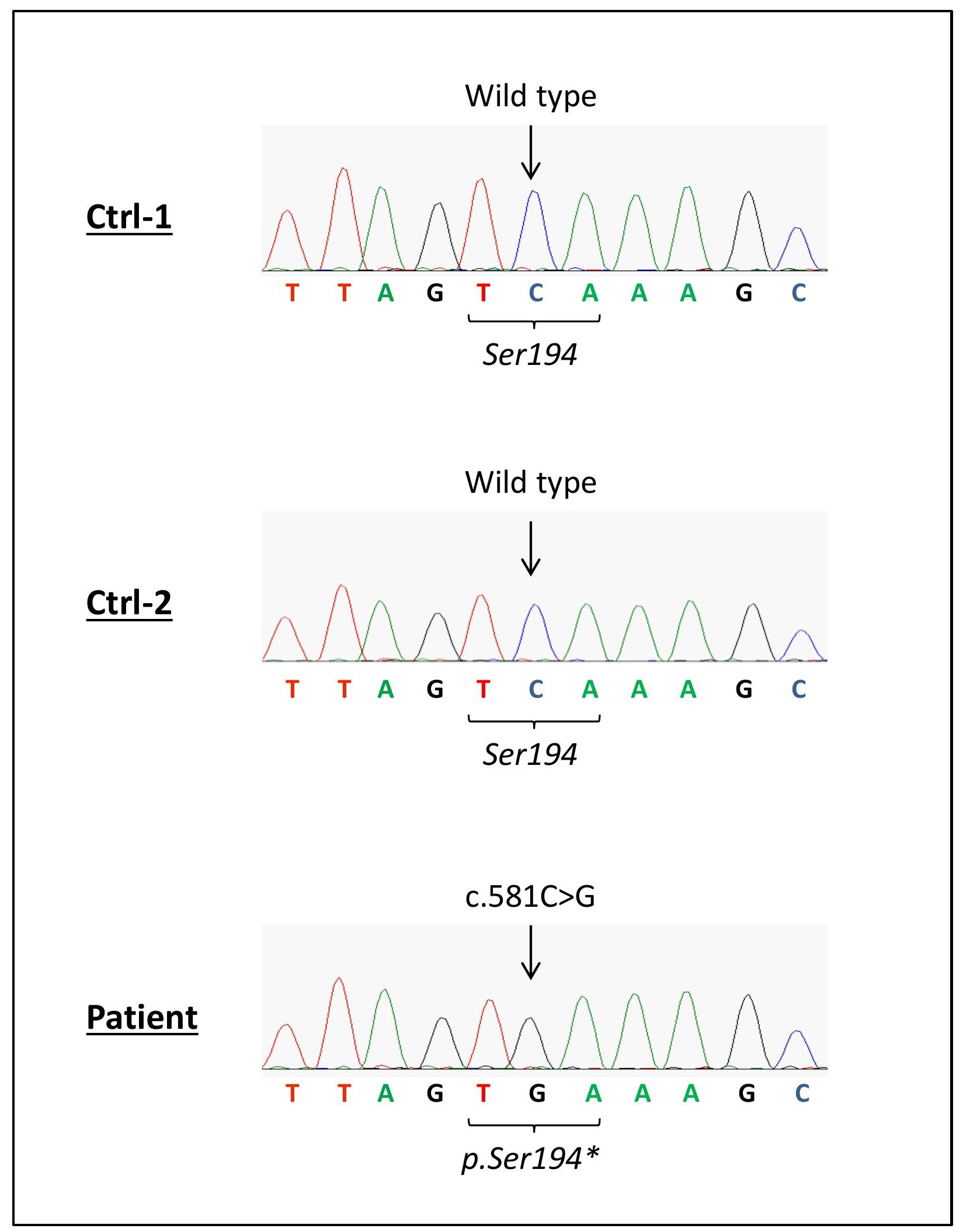

Supplement: Supplementary file 1 [file biomedicines-09-00945-s001.zip › Figure S1.jpg]

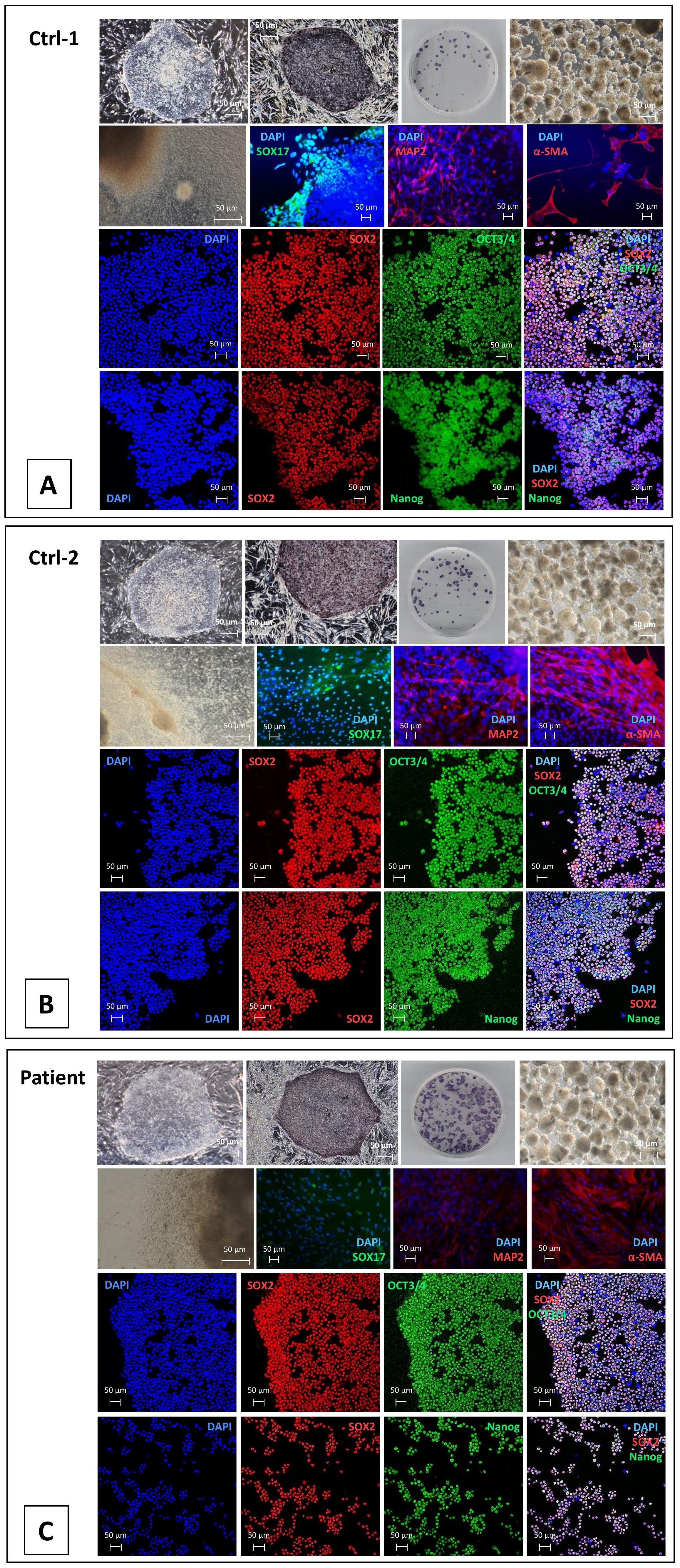

Supplement: Supplementary file 1 [file biomedicines-09-00945-s001.zip › Figure S2.jpg]

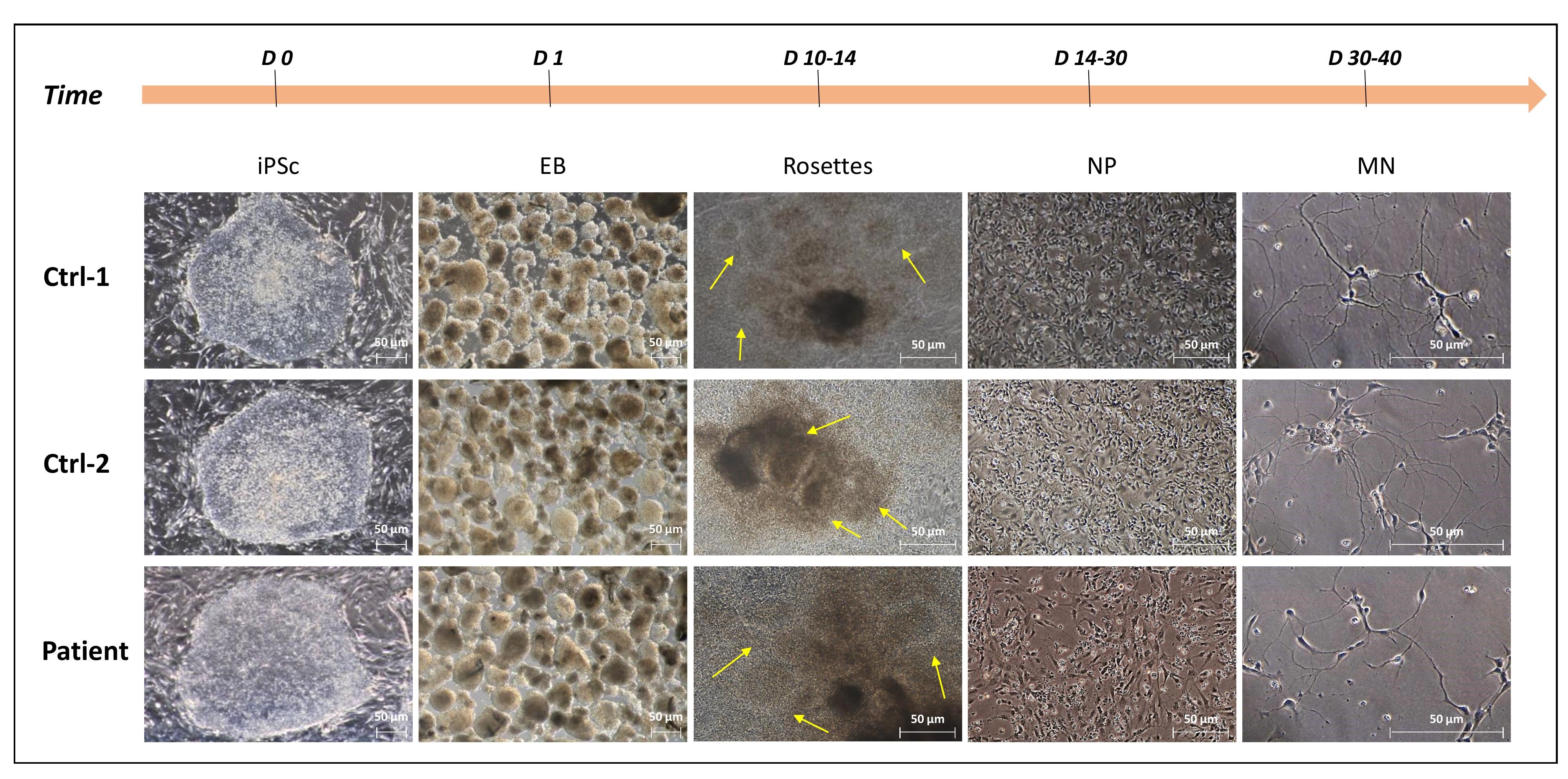

Supplement: Supplementary file 1 [file biomedicines-09-00945-s001.zip › Figure S3.jpg]

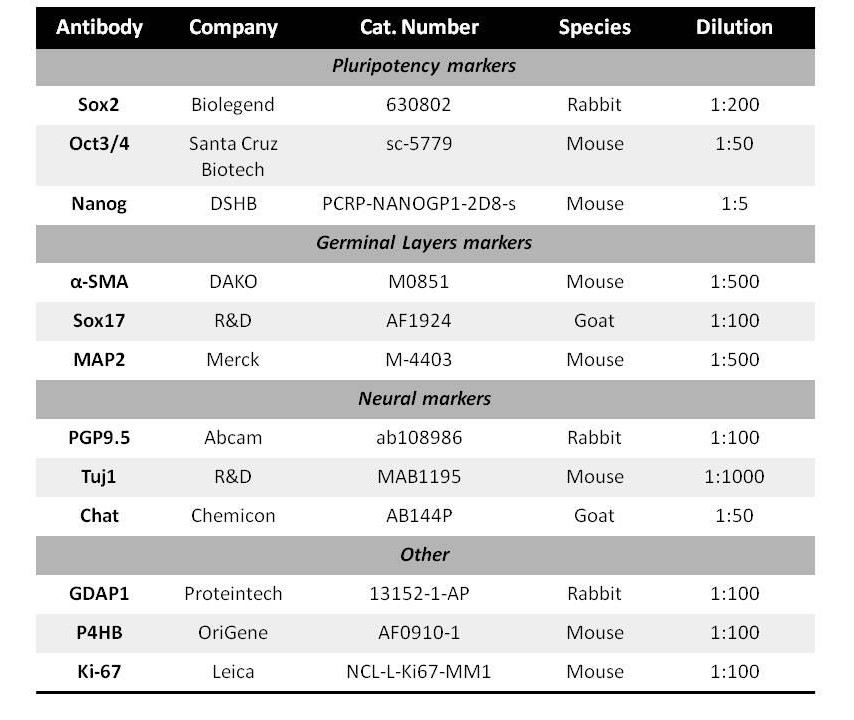

Supplement: Supplementary file 1 [file biomedicines-09-00945-s001.zip › Table S1.jpg]
